# Dermal fibroblast mitochondrial profiles in painful diabetic neuropathy

**DOI:** 10.1007/s00125-025-06660-8

**Published:** 2026-01-26

**Authors:** Julie Mie Mølgaard Bentzen, Peter Kolind Brask-Thomsen, Maiken Krogsbæk, Xiaoli Hu, Jens Randel Nyengaard, Sandra Sif Gylfadottir, Pall Karlsson, Nanna Brix Finnerup, Rikke Katrine Jentoft Olsen, Zahra Nochi

**Affiliations:** 1 Research Unit for Molecular Medicine, Department of Clinical Medicine, Aarhus University, Aarhus, Denmark; 2 Danish Pain Research Center, Department of Clinical Medicine, Aarhus University, Aarhus, Denmark; 3 Core Centre for Molecular Morphology, Department of Clinical Medicine, Aarhus University, Aarhus, Denmark; 4 Department of Pathology, Aarhus University Hospital, Aarhus, Denmark; 5 Department of Neurology, Aarhus University Hospital, Aarhus, Denmark

**Keywords:** Bioenergetics, Cytokine secretion, Dermal fibroblasts, Diabetic polyneuropathy, Fibroblast phenotyping, Immunohistochemistry, Macrophage infiltration, Mitochondrial function, Neuropathic pain, Skin biopsy

## Abstract

**Aims/hypothesis:**

Dermal fibroblasts have emerged as potential contributors to chronic pain, yet their role in diabetic polyneuropathy (DPN) and neuropathic pain remains poorly defined. Mitochondrial dysfunction and low-grade inflammation have been implicated in different pain conditions, but whether fibroblast mitochondrial health and cytokine secretion contribute to painful DPN is unknown.

**Methods:**

We conducted an integrated cellular and molecular profiling of dermal fibroblasts and skin biopsies from 30 participants, grouped into control participants (*n*=5), diabetes without DPN (*n*=7), pain-free DPN (*n*=7) and painful DPN (*n*=11). Fibroblast cultures (*n*=24) were evaluated for morphology, growth rate, phenotype, inflammatory mediator secretion and mitochondrial function. Immunohistochemistry of skin biopsies was used to assess fibroblast density, mitochondrial markers and immune cell infiltration.

**Results:**

Fibroblast morphology and proliferation did not differ significantly between groups. Flow cytometric profiling revealed no significant differences in fibroblast subtype distributions across groups. Inflammatory mediator secretion was limited. Mitochondrial mass, membrane potential, reactive oxygen species production and bioenergetic parameters were not different across groups. Skin biopsy analyses confirmed comparable fibroblast density and mitochondrial profiles across groups, regardless of neuropathy or pain. Notably, dermal macrophage infiltration was significantly elevated in the painful DPN group (mean ~8%; ANOVA *p*=0.02; painful DPN vs control participants *p*=0.02; painful DPN vs pain-free DPN *p*=0.07), consistent with prior findings from the same cohort, while Langerhans cell area fraction did not differ between groups.

**Conclusions/interpretation:**

Fibroblasts from participants with painful DPN did not differ in inflammatory and mitochondrial profiles compared with those from pain-free DPN. However, persistent dermal macrophage infiltration in painful DPN suggests a stable immune-activated microenvironment, potentially contributing to pain maintenance. Our results suggest that immune-related, rather than fibroblast-intrinsic, mechanisms could play a role in sustaining neuropathic pain in painful DPN.

**Graphical Abstract:**

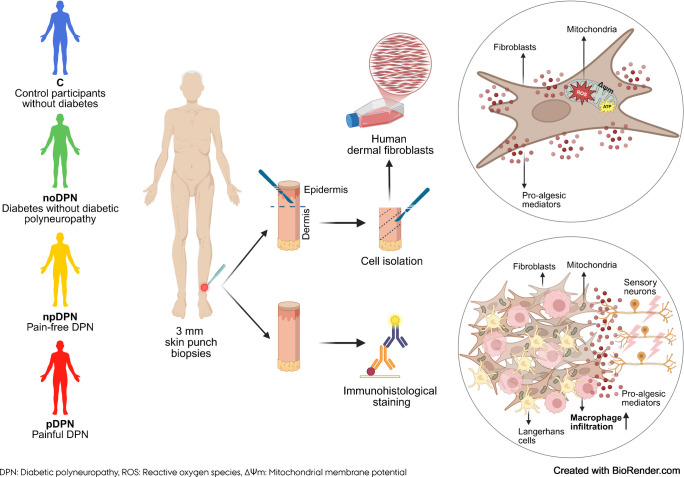

**Supplementary Information:**

The online version of this article (10.1007/s00125-025-06660-8) contains peer-reviewed but unedited supplementary material.


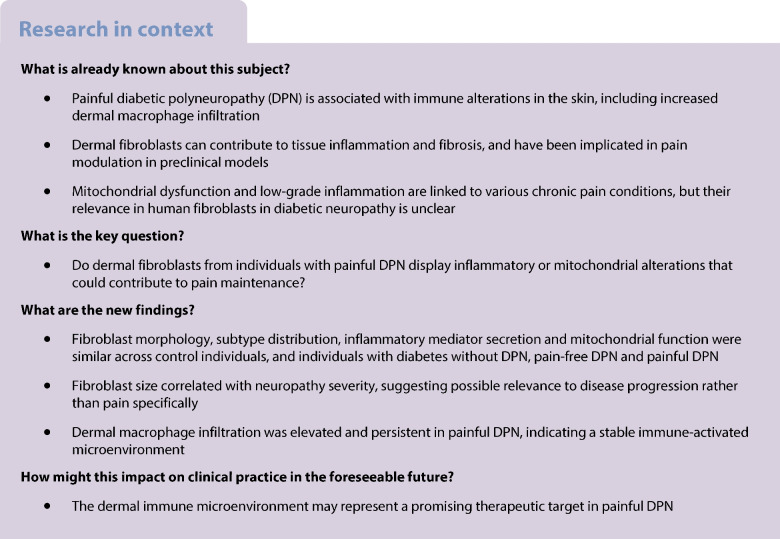


## Introduction

Neuropathic pain is a common and often debilitating condition caused by a lesion or disease affecting the somatosensory nervous system [1]. It is present in a wide range of diseases, including diabetic- and chemotherapy-induced neuropathy, peripheral nerve injury and radiculopathy [2–4]. Despite intensive research, current treatment strategies often provide only partial relief and are frequently associated with adverse effects [5], underscoring the urgent need for novel therapeutic targets.

Historically, neuropathic pain research has focused on neuronal mechanisms. However, accumulating evidence underscores the pivotal role of non-neuronal cells in modulating pain [6]. Among these, fibroblasts have emerged as promising yet underexplored contributors to chronic pain [7]. Fibroblasts are dynamic mesenchymal stromal cells that, beyond their classical role in extracellular matrix remodelling, actively participate in inflammation, fibrosis and tissue repair [8–10]. In response to disease-relevant stimuli such as hyperglycaemia, oxidative stress or tissue injury, they produce proinflammatory cytokines such as IL-6 and IL-1β and orchestrate immune cell recruitment by altering the extracellular environment [11]. In the dermis, fibroblasts contribute to both structural integrity and immune surveillance, recognising pathogen- and damage-associated molecular patterns (PAMPs and DAMPs) via pattern recognition receptors, and modulating local immune responses through cytokine and chemokine secretion [9].

Situated close to sensory nerve terminals, fibroblasts can influence peripheral sensitisation by releasing pain-relevant mediators, including IL-6, IL-1β and TNF-α [12–14]. Notably, recent work has demonstrated that activation of Toll-like receptor 4 on dermal fibroblasts is sufficient to induce and sustain pain-like behaviours in mice, highlighting their capacity to directly drive nociceptive signalling [15]. Beyond classical pathogen- or damage-associated stimuli, inflammation can also originate from endogenous stressors, notably mitochondrial dysfunction and the associated production of reactive oxygen species (ROS). Mitochondrial ROS, generated through electron transport chain leakage, can trigger intracellular signalling cascades that activate fibroblasts and induce proinflammatory mediator release, even in the absence of exogenous insults [16].

These insights are further enriched by growing interest in the role of mitochondria in chronic pain [17]. As central regulators of energy metabolism, redox balance and immune signalling [18–20], mitochondria are increasingly recognised as key modulators of pain pathophysiology. However, little is known about mitochondrial function in human skin fibroblasts in the context of pain.

In this exploratory study, we aimed to comprehensively characterise inflammatory profiles and mitochondrial function in primary dermal fibroblasts derived from a clinically well-defined cohort of participants with diabetic polyneuropathy (DPN), with and without pain, as well as control participants without diabetes. Fibroblast-secreted inflammatory mediators were profiled using multiplex assays, while mitochondrial integrity was systematically assessed through complementary analyses of mitochondrial bioenergetics, mass, membrane potential and superoxide production. In parallel, skin biopsies were analysed to quantify fibroblast density and mitochondria. To further explore local cutaneous immune activation, we quantified epidermal Langerhans cell area fraction and dermal macrophage infiltration (area fraction) by immunohistochemistry.

This integrated cellular and molecular characterisation aimed to uncover novel non-neuronal mechanisms contributing to the pathophysiology of painful DPN (pDPN). Our primary objective was to compare fibroblast–immune–mitochondrial features across four predefined clinical groups (control participants without diabetes, diabetes without DPN [noDPN], pain-free DPN [npDPN] and pDPN). By systematically investigating these previously underexplored cellular interactions, this study seeks to generate new hypotheses regarding peripheral drivers of neuropathic pain.

## Methods

### Standard protocol approvals, registrations and participant consents

All participants were provided with detailed oral and written information about the study prior to participation and signed an informed consent form before undergoing any examinations. The study was approved by the Regional Research Ethics Committee of the Central Denmark Region (1-10-72-130-16).

### Participants and clinical diagnosis

This study was part of a larger longitudinal investigation [21], including a follow-up of a previously characterised cohort of participants with type 2 diabetes mellitus and control participants without diabetes [22] recruited at Aarhus University Hospital between 2021 and 2023.

Participants were recruited from a well-characterised Danish cohort of individuals with type 2 diabetes originally enrolled through the nationwide Danish Centre for Strategic Research in Type 2 Diabetes (DD2) initiative, together with control participants without diabetes recruited from participants’ social networks and local advertisements. Control participants were recruited to be comparable to the diabetes group with respect to age and sex. Participants were not formally matched on continuous variables such as age; however, the groups were comparable with respect to age. The DD2 cohort has previously been shown to be broadly representative of adults with type 2 diabetes in Denmark, with no major differences in demographic or diabetes-related characteristics between participants who completed the clinical examination and those who did not. The source population reflects the demographics of the Central Denmark Region and is predominantly of White Northern European ethnicity (>95%), with limited ethnic diversity. In our subset (*N*=30), the distributions of age and sex were comparable to those of the source cohort. Education level was available for the original cohort and indicated somewhat lower representation of higher education among individuals with diabetes; no additional ethnicity or socioeconomic data were collected for the present study.

All participants underwent comprehensive clinical evaluations, including detailed pain history assessments, standardised neurological examinations, nerve conduction studies and intraepidermal nerve fibre density (IENFD) quantification, as previously described [21]. Detailed phenotyping included characterisation of neuropathic pain, pain descriptors and somatosensory profiles [21]. The severity of DPN was quantified using the Toronto Clinical Neuropathy Score (TCNS), and pain intensity over the preceding 7 days was evaluated using a Numeric Rating Scale (NRS).

Participants for this sub-study were selected based on predefined clinical criteria. Inclusion required either absence of DPN (noDPN) or definite DPN according to Toronto consensus criteria [23], further subclassified into npDPN and pDPN. All individuals with DPN had a confirmed diagnosis of neuropathy for more than 5 years. Participants with pDPN reported persistent, daily neuropathic pain for over 5 years, whereas those with npDPN exhibited neuropathy without current pain symptoms at the time of assessment. Individuals with significant comorbidities or other severe pain conditions were excluded. Skin biopsies were obtained by convenience sampling from participants from the ongoing follow-up study [21], prior to definitive group stratification. Inclusion was determined by biopsy availability and fibroblast viability. Control participants without diabetes were in the same manner selected based on biopsy availability.

### Skin biopsy

**Fig. 1:**
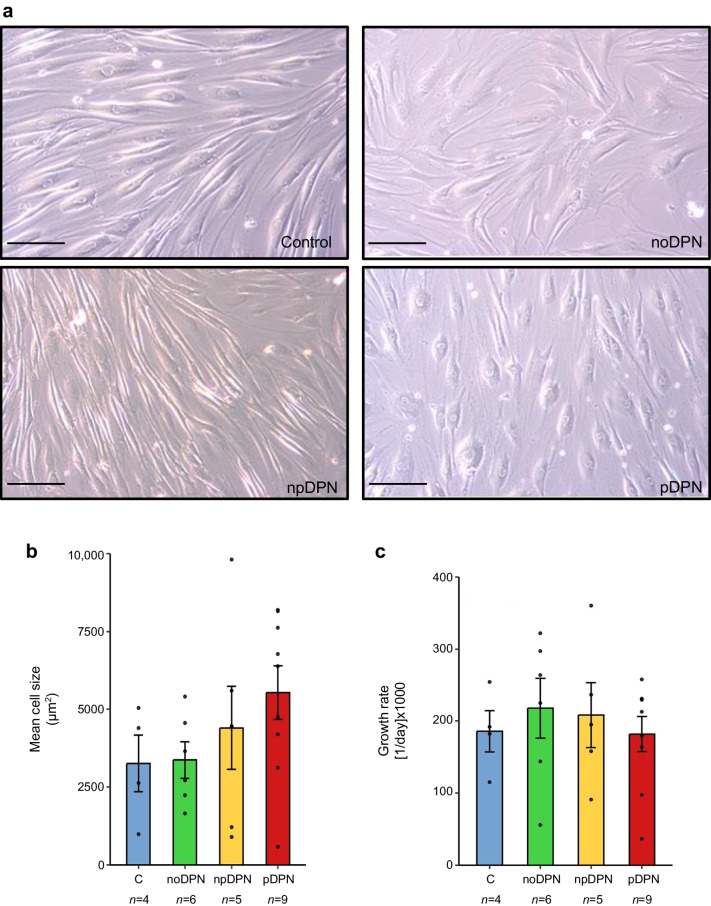
Morphology, size and proliferation of primary dermal fibroblasts derived from control participants without diabetes and participants with diabetes, with and without DPN and pain. (**a**) Images of cultured primary dermal fibroblasts derived from a control participant and participants with noDPN, npDPN and pDPN. Scale bars, 100 μm. (**b**) Quantitative analysis of mean fibroblast cell size (μm^2^) across groups shows no significant differences (ANOVA, *p*=0.3), despite a trend towards increased size in pDPN fibroblasts. (**c**) Fibroblast proliferation rates ([1/day] × 1000), measured by nuclear dye-based cytometry over 96 h, were comparable across groups (ANOVA, *p*=0.9). Bars represent mean ± SD; each dot represents one individual. C, control

Two 3-mm punch skin biopsies (Miltex) were collected from the lower leg of each participant, precisely 10 cm proximal to the lateral malleolus, under aseptic conditions and local anaesthesia using 1% lidocaine. One biopsy was transferred to 10 ml of RPMI-1640 medium with HEPES buffer, without l-glutamine (Thermo Fisher Scientific), supplemented with 10% FBS (Thermo Fisher Scientific), 1% penicillin/streptomycin (Euroclone) and nystatin (10,000 U/ml) (Thermo Fisher Scientific). This biopsy was then transported under controlled conditions and maintained at room temperature overnight for fibroblast culture establishment. The second biopsy was immediately fixed in Zamboni’s solution and cryoprotected in 20% sucrose overnight. The biopsies were subsequently snap frozen and stored at −20°C for immunohistological staining (electronic supplementary material [ESM] Fig. 1).

Although primary fibroblast cultures were successfully established from all 30 participants, six cell lines were irreversibly lost due to a freezer malfunction during storage. As a result, 24 fibroblast lines (control *n*=4; noDPN *n*=6; npDPN *n*=5; pDPN *n*=9) were available for subsequent experiments involving morphology, proliferation, flow cytometry, inflammatory profiling and mitochondrial function.

#### Primary fibroblast cultures

Skin biopsies were placed in a Petri dish with RPMI-1640 medium (supplemented with 10% FBS, 1% penicillin/streptomycin and 1% l-glutamine; Sigma-Aldrich). The epidermis and dermis were manually separated using No. 15 scalpels, then cut into ~16 uniform pieces, which were transferred into two T-25 cell culture flasks. To promote tissue attachment, 5 ml of culture medium was gently added while the flasks were held inverted, followed by overnight incubation at 37°C with 5% CO₂. The next day, the flasks were carefully returned to their upright position. Mycoplasma testing was performed 1 week after culture initiation, coinciding with the first medium change. Subsequent medium replacements were carried out every 3–4 days, based on visual assessment of fibroblast outgrowth under a light microscope (Carl Zeiss MicroImaging, 37081). Established primary skin fibroblasts were cryopreserved in a biobank at passage 3 or 4, once they reached 80% confluency. Experimental assays were performed on aliquots thawed from this early-passage biobank under uniform culture conditions across groups.

For all subsequent experiments, fibroblasts were cultured under identical conditions using low-glucose DMEM (5.5 mmol/l glucose) supplemented with 10% FBS, 1% penicillin/streptomycin and 1% l-glutamine. The use of low-glucose DMEM was intended to reflect a well-regulated diabetic metabolic state. All in vitro fibroblast assays were performed under basal culture conditions without exogenous inflammatory or metabolic stimulation.

#### *Cell morphology*

Fibroblast cell morphology was assessed from bright-field images acquired systematically using a Zeiss Axiocam 105 colour camera at ×20 magnification. In total, 25 fields were sampled from T-25 flasks at approximately 80% confluence.

Images were calibrated according to the embedded scale bar (pixels per µm) and imported into the newCAST software (v.2020.08.4.9377) (Visiopharm, Hørsholm, Denmark). Cell cross-sectional area was estimated using the unbiased 2D nucleator method as previously described in detail [24]. Briefly, the nucleator employs multiple radial test lines originating from a central reference point within each cell profile, recording intersections with the cell boundary to estimate cell area. In the current study, nine uniformly distributed test rays were applied to each fibroblast profile within a predefined counting frame. All cells fully inside the counting frame or intersecting the frame's inclusion borders were measured. This stereological approach provided unbiased and reproducible measurements of fibroblast cross-sectional area, enabling quantitative comparisons between experimental conditions.

#### *Cell growth rate*

Fibroblasts were cultured at approximately 80% confluence and harvested after 24, 48, 72 and 96 h of incubation. Following incubation, the growth medium was aspirated, and cells were washed twice with PBS (pH 7.4). Cells were then trypsinised, neutralised with fresh medium and centrifuged at 400 *g* for 5 min. The resulting cell pellet was resuspended in 1–3 ml of culture medium for counting.

For quantification, 100 µl of the suspension was mixed with 1 µl of DAPI solution (500 µg/ml; ChemoMetec) and 99 µl of lysis buffer (acidic surfactant solution, pH 2–3; ChemoMetec) to detect total cells, and with 1 µl of DAPI in 199 µl of PBS for dead cell detection. Fluorescence was measured using the NucleoCounter NC-3000 image cytometer (ChemoMetec).

The cell growth rate was calculated using the formula: Growth rate = log_*e*_(*N*_*T*1_ / *N*_*T*4_) / *h* × 24, where *N*_*T*1_ and *N*_*T*4_ represent cell counts at 24 h and 96 h, respectively, and *h* is the time interval between the two measurements. This provides the mean proliferation rate per hour, scaled to a daily growth rate and expressed as [1/day].

The experiment was conducted once, and growth rates were calculated for each cell line. The 24–96 h time window was selected to capture the optimal growth phase of fibroblasts, as previously described [25].

#### *Flow cytometry*

To investigate whether fibroblast heterogeneity differs across clinical groups, we performed multiparametric flow cytometry to profile dermal fibroblast subtypes using surface markers with known biological relevance. CD90 (also known as Thy-1) is a canonical fibroblast marker that distinguishes between developmentally distinct and functionally divergent fibroblast populations. CD26 (dipeptidyl peptidase-4) is associated with profibrotic activation and extracellular matrix remodelling, while CD39 (ectonucleoside triphosphate diphosphohydrolase-1 [ENTPD1]) modulates immune responses through ATP hydrolysis and contributes to an anti-inflammatory phenotype. Fibroblast activation protein (FAP) is expressed on activated fibroblasts and is often upregulated in tissue injury and inflammation [26].

To exclude contaminating cell types, we included CD45 (leukocyte common antigen) to exclude heamatopoietic cells, CD31 (platelet endothelial cell adhesion molecule-1 [PECAM-1]) to exclude endothelial cells and CD326 (epithelial cell adhesion molecule [EpCAM]) to exclude epithelial contamination.

Fibroblasts were harvested at approximately 80% confluency, washed in PBS, trypsinised, neutralised with fresh medium and centrifuged (400 *g*, 5 min). Cell pellets were resuspended in 1–3 ml of culture medium and counted. For antibody staining, single-cell suspensions (1.5×10^5^ cells/ml) were incubated at 4°C in the dark for 30 min with fluorochrome-conjugated antibodies targeting fibroblast, endothelial and leukocyte markers: anti-FAP APC (rabbit IgG, monoclonal; Nordic Biosite, bsm-60636R-APC-100), anti-CD90 Brilliant Violet 605 (mouse IgG1 κ, clone 5E10; Nordic Biosite, 328128), anti-CD26 phycoerythrin (PE) (mouse IgG2a κ, clone BA5b; Nordic Biosite, 302706), anti-CD36 FITC (mouse IgG2a κ, clone 5-271; Nordic Biosite, 336203), anti-CD45 Pacific Blue (mouse IgG1 κ, clone HI30; Nordic Biosite, 304054), anti-CD31 peridinin–chlorophyll–protein (PerCP)/Cyanine5.5 (mouse IgG1 κ, clone WM59; Nordic Biosite, 303106), anti-CD39 PE/ cyanine 7 (Cy7) (mouse IgG1 κ, clone A1; Nordic Biosite, 328212) and anti-CD326 (EpCAM) Brilliant Violet 711 (mouse IgG2b κ, clone 9C4; Nordic Biosite, 324240). A negative control without antibodies was included for each experiment.

Staining was conducted in a non-stick cell sorting buffer (PBS supplemented with 1% BSA, 2.5 mmol/l EDTA, 25 mmol/l HEPES; Core Facility for Reagents, Department of Biomedicine, Aarhus University, Denmark) to minimise cell adhesion. After staining, cells were washed twice with the same buffer and kept at 4°C in the dark until analysis, performed within the same day. Viable cells were identified using the viability stain Zombie NIR (Nordic Biosite, 423105).

Polychromatic flow cytometry was performed using a NovoCyte Penteon flow cytometer equipped with five lasers (349 nm, 405 nm, 488 nm, 561 nm and 637 nm) and 30 fluorescence detectors (Agilent). Cells were analysed at a core stream diameter of 16.7 µm and a flow rate of 65 µl/min. A cooling block was employed throughout the analysis to maintain cell suspension uniformity and minimise aggregation.

Spectral overlap between fluorophores was corrected using Compensation Beads (BioLegend) as single-stain controls. For the Zombie NIR viability dye, cell-based compensation was necessary; thus, cells were heat-treated (95°C, 3 min) to generate a distinct dead cell population. Compensation matrices were automatically calculated and applied using the built-in compensation function of the analysis software.

Data analysis was performed using FlowJo software (v10.10.0, Tree Star).

#### *Gating strategy*

Flow cytometry data were analysed using a hierarchical gating approach (detailed in ESM Fig. 2). Briefly, viable cells were identified by gating on low Zombie NIR fluorescence intensity combined with FSC-H. Singlet gating was subsequently performed by plotting FSC-A vs FSC-H. Lineage-negative cells (CD31⁻CD45⁻) were selected to exclude endothelial and leukocyte populations. These live lineage-negative fibroblasts were then classified based on CD90 expression into CD90⁺ and CD90⁻ subsets. Additionally, FAP expression was assessed and epithelial contamination (CD326⁺) was excluded. Finally, fibroblast subpopulations were identified based on the expression of CD326, FAP, CD90, CD26, CD36 and CD39, with gates consistently set relative to unstained controls.

### Inflammatory profiling

Primary fibroblasts (12,500 cells/well) were seeded into 24-well plates in standard culture medium and incubated at 37°C with 5% CO₂. After 24 h, cell attachment and morphology were confirmed microscopically, and the culture medium was replaced with serum-deprived DMEM (containing 1% FBS, 1% penicillin-streptomycin and 1% l-glutamine). Cells were incubated under serum-deprived conditions for an additional 24 h, after which conditioned cell supernatants were collected and centrifuged (400 *g*, 10 min, 4°C) to remove cellular debris.

Inflammatory cytokines (IL-1α, IL-1β, IL-2, IL-4, IL-6, IL-10, TNF-α) and the chemokine C-X-C motif chemokine ligand 8 (CXCL8) were quantified using a multiplex immunoassay (Human ProcartaPlex Multiplex Immunoassay; Invitrogen, Thermo Fisher Scientific) according to the manufacturer’s instructions. Samples were analysed on the MAGPIX instrument (Luminex XMAP Technologies). Cytokine concentrations below the assay detection limit were assigned random values between zero and the lower detection threshold, whereas measurements exceeding the assay’s upper detection limit were assigned the maximum detectable concentration.

### Mitochondrial function and bioenergetics

#### Mitochondrial mass

Mitochondrial mass was assessed using MitoTracker Green dye (Invitrogen, Thermo Fisher Scientific), a fluorescent stain localising specifically to mitochondria irrespective of membrane potential [27]. Fibroblasts (75,000 cells/well) were seeded into six-well plates in standard DMEM medium. After 24 h, adherence and uniform cell distribution were confirmed microscopically, and the medium was replaced with serum-deprived DMEM for an additional 24 h incubation. Cells were then stained with MitoTracker Green (100 nmol/l in Hanks’ balanced salt solution, 30 min at 37°C, protected from light). After staining, cells were gently washed with PBS, counterstained with Reddot2 (5 µmol/l, Biotium) to differentiate dead cells and analysed immediately using the NucleoCounter NC-3000 (ChemoMetec). Data were processed using NucleoView software (v.2.1.25.0) (ChemoMetec).

#### Mitochondrial membrane potential

Fibroblasts (1×10⁶ cells per condition) were seeded in standard DMEM and incubated for 24 h, after which the medium was changed to serum-deprived DMEM for an additional 24 h incubation. Cells were then stained with 5,5′,6,6′-tetrachloro-1,1′,3,3′-tetraethylbenzimidazolylcarbocyanine iodide (JC-1) dye (200 µg/ml, ChemoMetec) for 15 min at 37°C under gentle agitation. Carbonyl cyanide m-chlorophenyl hydrazone (CCCP, 50 µmol/l) served as a positive control for mitochondrial depolarisation [28]. Following staining, cells were washed twice with PBS, counterstained with DAPI (1 µg/ml, ChemoMetec) and analysed immediately using the NucleoCounter NC-3000. The gating strategy was guided by CCCP-treated control samples. Data were analysed using NucleoView software.

#### Mitochondrial superoxide

Fibroblasts (150,000 cells/well) were seeded into six-well plates and incubated at 37°C with 5% CO₂. After verifying cell attachment at 24 h, mitochondrial superoxide production was assessed using mitochondrial superoxide indicator (MitoSOX Red dye; 5 µmol/l, Invitrogen, Thermo Fisher Scientific). Cells were stained for 20 min at 37°C following a 10 min pre-treatment with antimycin A (150 µmol/l), a mitochondrial ROS inducer used as a positive control [29]. Cells were subsequently washed with PBS, harvested and counterstained with Hoechst 33342 (10 µg/ml, Thermo Fisher Scientific) and Reddot2 (300 nmol/l, Biotium). Analysis was conducted immediately using the NucleoCounter NC-3000, with gating established based on positive control populations.

#### Mitochondrial bioenergetics

Mitochondrial bioenergetic profiles were determined using a Seahorse XFe96 Analyzer (Agilent Technologies). Fibroblasts were seeded at a density of 12,000 cells per well into XF96 microplates and cultured for 24 h in standard DMEM. Following confirmation of equal cell attachment, the medium was changed to serum-deprived DMEM, and cells were cultured for an additional 24 h. On the assay day, the medium was replaced with Seahorse XF assay medium (XF HEPES medium, pH 7.4, supplemented with 10 mmol/l glucose, 1 mmol/l sodium pyruvate and 2 mmol/l glutamine). Oxygen consumption rate (OCR) and proton efflux rate (PER) were measured after sequential injections of oligomycin (1.26 µmol/l), FCCP (1 µmol/l) and rotenone/antimycin A (each 0.5 µmol/l). Each injection was followed by triplicate measurements.

OCR and PER were normalised to cell counts obtained after nuclear staining with Hoechst 33342 (4 µmol/l, Thermo Fisher Scientific) using automated imaging by Cytation 1 (BioTek, Winooski, VT, USA). Bioenergetic parameters were calculated using Wave software (v.2.6.3) (Agilent Technologies).

### Immunohistological staining

#### Tissue preparation and staining procedures

Skin biopsies were processed for immunohistochemistry and immunofluorescence staining to assess intraepidermal nerve fibres, fibroblasts, mitochondrial markers, macrophages and Langerhans cells.

Staining for intraepidermal nerve fibres for the estimation of IENFD was performed using the standardised protocol for free-floating sections using the immunoperoxidase method, as described in detail elsewhere [30, 31].

Cryostat sections (10 µm, Cryostar NX70) were stained for fibroblast and mitochondrial markers using: fibroblast-specific protein 1 (FSP1) (1:250, 07-2274, Sigma-Aldrich), heat shock protein 60 (HSP60) (1:25, Ab110312, Abcam) and translocase of the outer mitochondrial membrane 20 (TOMM20) (1:300, Ab186735, Abcam). Three sections per participant were blocked in 1% BSA in PBS with Tween-20 (PBST; 0.3% Triton X-100) and incubated overnight with primary antibodies. Sections were either stained with a combination of FSP1 and HSP60, or with TOMM20, followed by an additional FSP1 staining after serum blocking. Secondary antibodies included: AF594 donkey anti-mouse (1:700, Abcam), AF594 donkey anti-rabbit (1:500, Abcam) or AF647 donkey anti-rabbit (1:600, Invitrogen). After PBS washes, sections were counterstained with DAPI (1:10,000 in PBS, Sigma) and mounted using fluorescent mounting medium (F4680, Sigma-Aldrich).

Langerhans cells and macrophages were visualised using immunofluorescence staining. Skin sections (50 µm thick) were collected in neutral PBS, treated with sodium citrate buffer at 70°C for 20 min and blocked with 1% normal donkey serum and 0.3% Triton X-100 in PBS for 1 h. Primary antibody incubation was performed overnight using: ionised calcium-binding adaptor molecule 1 (Iba1) (1:500; Wako Chemicals) for dermal macrophages and S100 (ready to use, DAKO) for epidermal Langerhans cells. Sections were incubated with secondary antibodies for 90 min: AF488 donkey anti-rabbit (1:700, Abcam) and AF647 donkey anti-rabbit IgG (H+L) (1:650, Invitrogen). DAPI (1:5000 in PBS, Sigma) was applied for nucleus counterstaining before mounting.

#### Imaging and quantification

IENFD was estimated by counting the number of nerve fibres crossing the epidermal–dermal border, divided by the section’s length, following published guidelines [30], and overall results were published previously [21].

#### *Fibroblasts and mitochondria*

Sections stained with FSP1 and HSP60 were scanned using a VS200 Olympus slide scanner (×100 oil) and analysed in QuPath (v.0.5.1, https://qupath.github.io/). The epidermis, subepidermal area (150–200 µm below the epidermis) and dermis were delineated. An autofluorescence-based threshold was applied (HSP60: 500, FSP1: 3500) and positive cells were quantified relative to total cell number (DAPI quantification). Sections stained with FSP1 and TOMM20 were imaged using a Zeiss LSM800 confocal microscope, capturing the epidermis and subepidermal area. Mitochondrial quantification was performed using the MitochondriaAnalyzer plugin in ImageJ/Fiji (downloaded 17 December 2024 from https://github.com/AhsenChaudhry/Mitochondria-Analyzer), measuring mitochondrial number and morphology both in total within the epidermis and subepidermal area, and specifically within FSP1-positive cells in the subepidermal region (ESM Fig. 3).

#### *Macrophages*

Iba1-positive macrophages (area fraction) were quantified using ImageJ (NIH and LOCI, WI, ImageJ2 2.16.0/1.54p; Java 1.8.0_322 [amd64]) from sections imaged on an Olympus BX51 fluorescence microscope. An RGB intensity threshold was applied to exclude background staining. The fluorescent area in the epidermal–dermal junction (up to 500 μm below) was measured and expressed as a percentage of the total region of interest [32, 33]. This subset of participants (*N*=30) was originally included in the baseline study by Gylfadottir et al (2022) [32], where dermal macrophage area fraction was assessed using identical staining and quantification protocols. In the present follow-up, we re-analysed new biopsies from the same anatomical site to evaluate whether macrophage infiltration persists over time.

#### *Langerhans cells*

Confocal images (15 stacks, 1.5 µm stack interval) were acquired using a Zeiss LSM800 confocal microscope (×40). Langerhans cell expression was measured by area fraction in the epidermis, using Fiji to orthogonally project confocal stack images into two dimensions [33]. The Langerhans cell area fraction was calculated as Langerhans cells divided by the total epidermal area. Comparisons of Langerhans cell area fraction between the four clinical groups will be reported separately.

### Statistical analysis

All statistical analyses were performed in R (v4.4.1) using RStudio (https://mirrors.dotsrc.org/cran/). This was an exploratory study aiming to characterise differences among the four clinical groups. Analyses followed a prespecified four-group design to separate neuropathy and pain phenotypes.

The distribution of continuous variables was assessed using the Shapiro–Wilk test and visual inspection of Q–Q plots. For normally distributed variables with homogeneous variances (assessed via Levene’s test), one-way ANOVA was applied. For non-normally distributed data or when assumptions of homogeneity were violated, the Kruskal–Wallis rank sum test was used.

For datasets containing repeated technical replicates (e.g. cell culture experiments), two-way mixed-effects ANOVA models were employed to account for both technical variability and biological variation. In these models, biological replicates (individual participant-derived fibroblast cultures) were included as random effects, while experimental group was treated as a fixed effect. This hierarchical structure ensured robust inference while preserving participant-level granularity. When appropriate, participant-level means were calculated for visualisation purposes.

Post hoc pairwise comparisons were performed when significant group-level effects were observed, using Tukey’s honestly significant difference (HSD) test for parametric data and Dunn’s test with Benjamini–Hochberg correction for non-parametric comparisons. Unless otherwise specified, data are presented as mean ± SD. A two-tailed *p* value <0.05 was considered statistically significant.

## Results

### Participant characterisation

A total of 30 participants were included: control, *n*=5; noDPN, *n*=7; npDPN, *n*=7; and pDPN, *n*=11. Among the 11 participants classified as pDPN, two reported dysaesthesia without pain. Clinical characteristics of the participants are summarised in Table 1.

**Table 1:** Clinical characteristics of participants

Characteristic	Control	noDPN	npDPN	pDPN
Sex, female	3 (60.0)	5 (71.4)	2 (28.6)	5 (45.5)
Participants (*N*=30)	*n*=5	*n*=7	*n*=7	*n*=11
Age (years)	66.6±12.0	73.0±4.7	74.1±5.2	69.3±10.2
BMI (kg/m^2^)	26.5±4.9	29.6±4.8	29.9±4.1	32.4±6.1
HbA_1c_ (mmol/mol)	37.2±4.1	52.9±15.5	45.3±6.2	50.6±12.6
HbA_1c_ (%)	5.6±0.4	7.0±1.4	6.3±0.6	6.8±1.2
Time since diabetes diagnosis (years)	NA	12.7±3.8	10.9±1.4	11.1±2.1
TCNS (0–19)	4.6±3.2	4.1±2.2	7.4±3.4	10.3±1.9
NRS (0–10)	NA	NA	NA	4.9±2.2
IENFD (fibres/mm)	7.3±3.6^a^	7.2±2.2	2.7±1.6	2.5±2.4
CRP (mg/l)	2.3±1.1	8.8±17.0	1.3±0.6	3.8±4.2
Leukocytes (10^9^/l)	5.8±0.9	8.1±1.8	8.8±1.1	8.9±2.4

Data are shown as *n* (%) or mean ± SD

Clinical characteristics of participants categorised into four groups: control participants, noDPN, npDPN and pDPN

^a^Control participant IENFD values fall within age-adjusted normative ranges [31]

NA, not applicable

Glycaemic control (HbA_1c_) and BMI were higher in all diabetic groups compared with control participants. Neuropathy severity (TCNS) increased progressively across groups, with the highest scores in pDPN. IENFD was markedly reduced in both DPN groups. In line with published normative reference values [31], the mean IENFD in our control group (7.3±3.6 fibres/mm, mean age 66.6 years) is within the expected normal range for this age. Systemic inflammatory markers showed group-related differences. Mean C-reactive protein (CRP) levels were highest in the noDPN group (8.8±17.0 mg/l), but this was not statistically significant (Kruskal–Wallis, *p*=0.10). In contrast, leukocyte counts differed significantly among groups (Kruskal–Wallis, *p*=0.02), with post hoc analyses indicating elevated leukocyte levels in the npDPN (*p*=0.04) and pDPN (*p*=0.02) groups compared with non-diabetic control participants.

### Fibroblast morphology and proliferation

#### Fibroblast morphology and size

To provide an initial visual impression of cellular phenotype, bright-field images of fibroblasts from each group are shown (Fig. 1a). These images suggest subtle morphological differences, such as a more spread-out appearance in fibroblasts from participants with pDPN. However, due to substantial inter-individual variability in cell size and morphology (ANOVA on participant-level data, *p*<2.2×10⁻^16^; ESM Fig. 4), these images should be viewed as illustrative examples rather than representative of each group.

Quantitative morphometric analysis of fibroblast cross-sectional area revealed no statistically significant differences between groups (ANOVA, *p*=0.30; Fig. 1b). Nonetheless, fibroblasts from both npDPN (5070±1481 μm^2^) and pDPN (5839±1711 μm^2^) participants showed numerically larger mean cell size compared with those from control (4574±1645 μm^2^) and noDPN (4135±1321 μm^2^) groups. This raises the possibility that morphological variation might be related to neuropathy rather than to pain. In line with this, there was a statistically significant group × TCNS interaction (*p*<0.0001), indicating a relationship between neuropathy severity and fibroblast size.

#### Fibroblast growth rate

Mean growth rates ranged from 177±44 × 10^3^ day⁻^1^ (control) to 220±65 × 10^3^ day⁻^1^ (noDPN), with no statistically significant differences observed between the clinical groups (ANOVA, *p*=0.90; Fig. 1c). These results suggest comparable proliferative capacity among fibroblast cultures under standardised conditions.

### Flow cytometric profiling of fibroblast subtypes

To explore whether fibroblast heterogeneity differed across clinical groups, we profiled subpopulations based on markers with distinct biological functions. CD90 (Thy-1) defined two major fibroblast populations (CD90⁺ and CD90⁻), while CD26 and CD39 provided further insight into fibrotic and anti-inflammatory potential, respectively. FAP expression was assessed as a marker of fibroblast activation but was undetectable across all samples.

Fibroblast cultures showed consistently high purity (>98% CD45⁻CD31⁻), with negligible epithelial contamination (CD326⁺). These results confirm the phenotypic identity and purity of isolated fibroblasts regardless of clinical status. Subtype analysis based on CD26 and CD39 expression revealed comparable distributions of CD90⁺ (Kruskal–Wallis, *p*=0.50) and CD90⁻ fibroblast subsets (*p*=0.50) across clinical groups. At the subtype level, no significant group differences emerged for any CD26/CD39-defined fibroblast populations (all *p*>0.07), despite notable inter-individual variability, particularly within the pDPN group (ESM Fig. 5). Collectively, these data indicate that while substantial individual variation exists, there were no significant group-level changes in fibroblast subtype distributions associated with diabetic neuropathy or neuropathic pain.

### Inflammatory profiling of fibroblasts

Baseline secretion of inflammatory mediators from participant-derived fibroblasts was assessed using a multiplex Luminex immunoassay. Among the analysed cytokines (IL-1α, IL-1β, IL-2, IL-4, IL-6, IL-10, TNF-α and CXCL8), only IL-6 and CXCL8 were reliably detectable across samples; the remaining mediators were consistently below the assay's lower detection threshold.

**Fig. 2:**
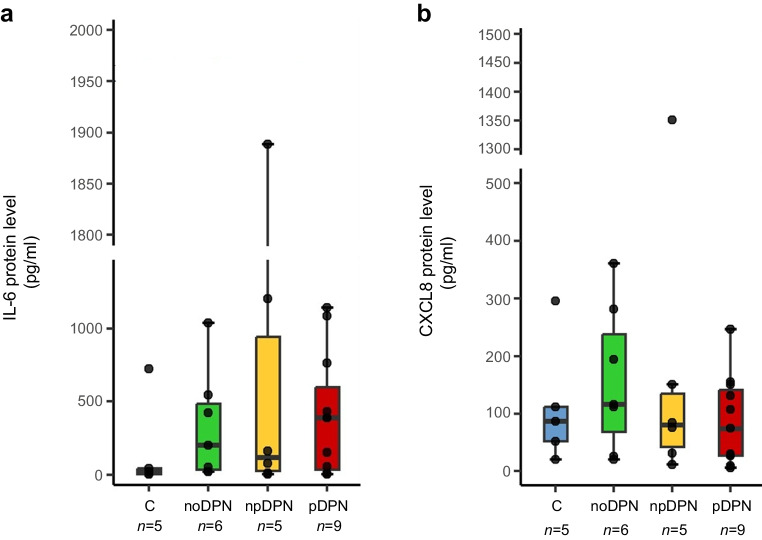
Basal secretion of inflammatory mediators IL-6 and CXCL8 by primary dermal fibroblasts from control participants without diabetes and participants with diabetes, with and without DPN and pain. Primary dermal fibroblasts were cultured under serum-deprived conditions, and basal secretion of IL-6 (**a**) and CXCL8 (**b**) in supernatants was quantified by multiplex immunoassay after 24 h of incubation. Statistical analysis was performed using Kruskal–Wallis rank sum test. No significant differences were identified among groups. Boxplots represent median ± IQR; whiskers represent minimum and maximum values; and each dot represents one individual. C, control

No significant differences were observed in IL-6 (Kruskal–Wallis, *p*=0.60; Fig. 2a) or CXCL8 (Kruskal–Wallis, *p*=0.90; Fig. 2b) secretion across participant groups. Collectively, these findings indicate only modest basal inflammatory activation of fibroblasts across all study groups, independent of diabetic and neuropathic status.

### Mitochondrial function and bioenergetics in fibroblasts

#### Mitochondrial mass, membrane potential and superoxide production

**Fig. 3:**
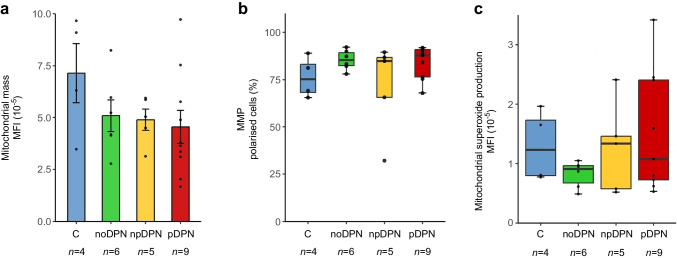
Mitochondrial integrity and oxidative stress in primary dermal fibroblasts from control participants without diabetes and participants with diabetes, with and without DPN and pain. (**a**) Mitochondrial mass was quantified using MitoTracker Green; no significant group differences were observed (ANOVA, *p*=0.3). Bars represent mean ± SD; each dot represents one individual. (**b**) MMP was assessed using JC-1 dye. The percentage of polarised cells was comparable across groups (Kruskal–Wallis, *p*=0.4). (**c**) Mitochondrial superoxide production, measured by MitoSOX Red fluorescence, did not differ significantly between groups (Kruskal–Wallis, *p*=0.6). (**b**, **c**) Boxplots represent median ± IQR; whiskers represent minimum and maximum values; and each dot represents one individual. C, control; MFI, mean fluorescence intensity

Mitochondrial mass, assessed via MitoTracker Green fluorescence, showed no statistically significant differences across groups (ANOVA, *p*=0.30; Fig. 3a), while control fibroblasts displayed slightly higher mean values, inter-individual variability was substantial across all conditions.

Mitochondrial membrane potential (MMP), evaluated by JC-1 staining, likewise demonstrated no group differences in the proportion of polarised cells (Kruskal–Wallis, *p*=0.40; Fig. 3b).

Superoxide production, measured with MitoSOX Red, did not differ significantly across the four groups (Kruskal–Wallis, *p*=0.60; Fig. 3c).

Collectively, these parameters indicate no significant alterations in basal mitochondrial health across the study groups.

#### Mitochondrial bioenergetics in fibroblasts

**Fig. 4:**
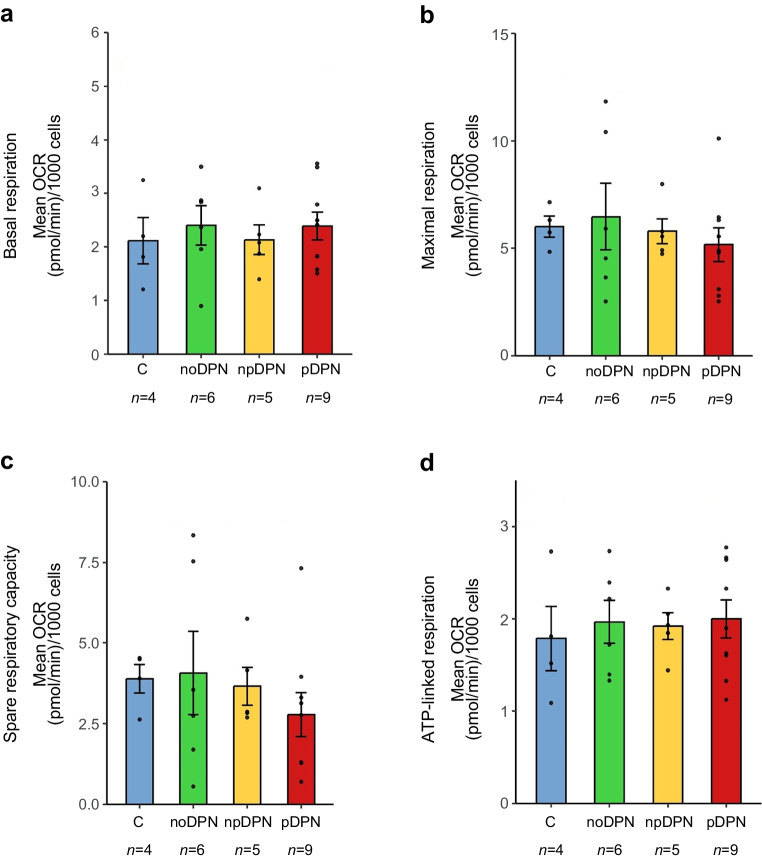
Bioenergetic profiling of primary dermal fibroblasts derived from control participants without diabetes and participants with diabetes, with and without DPN and pain. (**a**) Basal respiration, (**b**) maximal respiration, (**c**) spare respiratory capacity and (**d**) ATP-linked respiration were quantified by measuring OCR in fibroblasts derived from control participants without diabetes (C) and noDPN, DPN and pDPN groups. No significant differences were observed across groups (ANOVA, all *p*>0.6). Bars represent mean ± SD; each dot represents one individual. C, control

To assess mitochondrial respiratory function, we analysed OCR and PER in primary dermal fibroblasts across clinical groups using Seahorse XF analysis. No significant group differences were observed in basal respiration (ANOVA, *p*=0.90), maximal respiration (*p*=0.80), spare respiratory capacity (*p*=0.70) or ATP-linked respiration (*p*=0.90) (Fig. 4a–d). Likewise, extracellular acidification profiles, including basal and maximal PER as well as glycolytic reserve capacity, did not differ significantly between groups (ESM Fig. 6). These findings indicate preserved mitochondrial and glycolytic bioenergetic capacity in fibroblasts, regardless of neuropathy or pain status.

Assay responsiveness was verified in every run by the positive controls described in the Methods, which produced the expected responses.

### Fibroblast density and mitochondrial features in skin biopsies

We quantitatively characterised dermal fibroblast density, fibroblast mitochondrial profiles and epidermal mitochondrial expression across the groups.

#### Fibroblast density and morphology

Dermal fibroblast profile density, quantified as FSP1⁺ cells per tissue area, showed no significant differences among groups (one-way ANOVA, *p*>0.05) (ESM Fig. 7a). Similarly, mean fibroblast surface area measurements were comparable across all groups, indicating no consistent morphological changes related to diabetic neuropathy or pain status (one-way ANOVA, *p*>0.05) (ESM Fig. 7b).

#### Dermal and epidermal mitochondrial markers

We next assessed mitochondrial density and distribution by quantifying mitochondrial HSP60 and TOMM20.

#### *Dermis*

The density of HSP60⁺ cell profiles per tissue area in the dermal layers did not differ significantly among groups (one-way ANOVA, *p*>0.05) (ESM Fig. 7c). Analysis of fibroblast-specific mitochondrial profile density, defined as cells co-expressing HSP60 and FSP1 relative to total dermal cell profiles (DAPI), also showed no significant group differences (one-way ANOVA, *p*>0.05) (ESM Fig. 7d). Furthermore, the TOMM20 mitochondrial count per fibroblast and mitochondrial area per fibroblast area remained comparable across groups (one-way ANOVA, *p*>0.05 for both) (ESM Fig. 7e, f), suggesting no significant mitochondrial perturbations in dermal fibroblasts associated with neuropathy or pain.

#### *Epidermis*

Epidermal mitochondrial profile density, measured by HSP60⁺ cells per epidermal area, exhibited high variability within groups but did not significantly differ between groups (one-way ANOVA, *p*>0.05) (ESM Fig. 7g).

Taken together, our comprehensive analysis indicates no significant differences in dermal fibroblast density, morphology and mitochondrial markers in diabetic neuropathy, regardless of pain phenotype.

### Cutaneous immune cell infiltration

#### Dermal macrophage infiltration

**Fig. 5:**
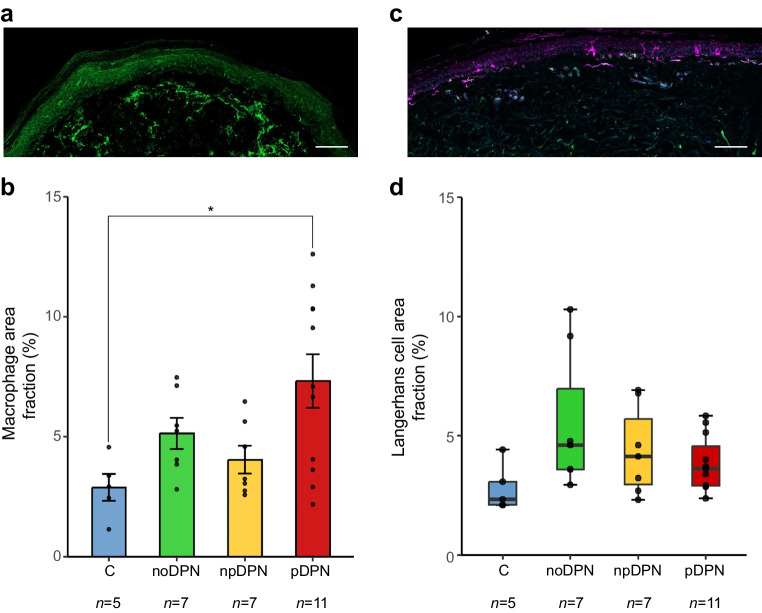
Immune cell profile densities in skin biopsies from control participants without diabetes and participants with diabetes, with and without DPN and pain. (**a**) Representative immunofluorescence image of Iba1-positive dermal macrophages (green). Scale bar, 100 μm. (**b**) Dermal macrophage area fraction density (% of total nucleated area fraction) was significantly elevated in pDPN compared with control participants (*p<0.05, one-way ANOVA with Tukey’s post hoc test). Bars represent mean ± SD; each dot represents one individual. (**c**) Representative immunofluorescence image of epidermal Langerhans cells (magenta). Scale bar, 100 μm. (**d**) Epidermal Langerhans cell area fraction (% of epidermal area) did not differ significantly between groups (Kruskal–Wallis, *p*=0.1), although a trend was observed. Boxplot represents median ± IQR; whiskers represent minimum and maximum values; and each dot represents one individual. C, control

Quantitative immunohistochemical analysis revealed significant differences in dermal macrophage area fraction across groups (one-way ANOVA, *p*=0.02). The pDPN group exhibited the highest macrophage infiltration, with a mean density of ~8% (Fig. 5a, b). Post hoc Tukey tests showed significantly higher macrophage area fraction in pDPN vs control participants (*p*=0.02). No other comparisons reached significance after adjustment.

#### Langerhans cell area fraction

Langerhans cell area fraction expression, assessed by area fraction in the epidermis, did not differ significantly between groups (Kruskal–Wallis, *p*=0.10) (Fig. 5c, d).

In a post hoc sensitivity analysis, we pooled the neuropathic groups (npDPN + pDPN) and contrasted them separately with control participants and with noDPN; no additional statistically significant differences were detected across any predefined endpoint (all *p*≥0.09; full outputs available on request).

## Discussion

This study provides the first comprehensive analysis of primary dermal fibroblasts in diabetic individuals with and without neuropathic pain, integrating morphometric, immunophenotypic, inflammatory and mitochondrial assessments both in vitro and in situ. While accumulating evidence has highlighted the relevance of non-neuronal contributors to neuropathic pain pathophysiology, particularly in inflammatory and fibrotic contexts [6], our findings do not support a prominent role for fibroblast-derived inflammation or mitochondrial dysfunction in the maintenance of pDPN. Instead, our results point to persistent dermal macrophage infiltration as the most consistent non-neuronal correlate of the painful phenotype. In addition, we observed that fibroblast size correlated with neuropathy severity, raising the possibility that morphological alterations in fibroblasts may be more relevant to the development or progression of neuropathy per se rather than to pain. The biological significance of fibroblast enlargement remains uncertain. It may reflect altered cellular homeostasis or increased protein synthesis; it may also reflect senescence-associated cell spreading, characterised by enlarged, flattened morphology rather than true hypertrophy and accompanied by a proinflammatory senescence-associated secretory phenotype (SASP) and impaired tissue repair [34, 35]. Because we did not measure canonical senescence markers, we cannot distinguish these possibilities. We also did not assess ultrastructural features (e.g. mitochondrial morphology or collagen bundles), which could provide additional insight. Future work should incorporate longitudinal sampling and targeted ultrastructural and functional analyses (e.g. cytoskeletal/extracellular matrix remodelling and mitochondrial ultrastructure), together with senescence phenotyping, to determine whether enlargement predicts neuropathy progression or represents secondary adaptation.

Fibroblasts have recently emerged as versatile regulators of tissue homeostasis and inflammation, including in the peripheral nervous system [8–10]. In mouse models, dermal fibroblasts can be activated via Toll-like receptor signalling to secrete pain-relevant cytokines such as IL-6 and IL-1β, thereby driving nociceptive sensitisation [15]. Given their anatomical proximity to nociceptive terminals and their capacity for long-term activation, fibroblasts are increasingly viewed as potential amplifiers of chronic pain states [36]. However, the current data do not indicate overt fibroblast activation in pDPN. Neither fibroblast density nor morphology in skin biopsies differed between groups, and in vitro assessments showed no group-level differences in cell size, proliferation or inflammatory cytokine secretion. While fibroblast size correlated with neuropathy severity in a group-specific manner, these patterns did not clearly map onto pain status, suggesting that fibroblast morphology may be modulated by neuropathy-related changes rather than pain per se.

Similarly, flow cytometric profiling of canonical fibroblast subtypes revealed no significant group differences. Previous studies have linked CD90 and CD26 expression with fibrogenic and inflammatory responses in skin fibrosis and arthritis [37–39], while CD39 marks fibroblasts with immunoregulatory capacity via adenosine signalling [40]. Despite substantial inter-individual heterogeneity, particularly in the pDPN group, no consistent shifts in these fibroblast subtypes were observed across clinical phenotypes. These findings extend recent single-cell transcriptomic studies suggesting that fibroblast subtype plasticity may depend on tissue context and inflammatory cues [38, 41].

Fibroblasts across all groups displayed comparable mitochondrial mass, membrane potential and superoxide production, and Seahorse analyses confirmed preserved mitochondrial respiration and glycolytic capacity, arguing against overt mitochondrial dysfunction in participant-derived fibroblasts. This contrasts with emerging literature in chronic pain and metabolic diseases, where mitochondrial dysfunction in sensory neurons or immune cells has been implicated in pain persistence [17, 42–45]. Notably, the use of low-glucose culture conditions may have masked latent metabolic vulnerabilities, as fibroblasts derived from individuals with diabetes often retain a degree of metabolic flexibility [46, 47]. Nonetheless, our data suggest that fibroblasts, unlike neurons or macrophages, do not exhibit overt mitochondrial dysfunction in pDPN.

In contrast to the largely negative findings for fibroblasts, dermal macrophage area fraction was significantly elevated in pDPN, replicating and extending prior observations [32]. Importantly, these findings replicate earlier observations in the same cohort from Gylfadottir et al (2022) [32], where mean macrophage area fraction density was ~8.0% in pDPN, compared with 5.1% in npDPN and 3.1% in control participants. In the current study, macrophage infiltration remains elevated approximately 5 years later, suggesting persistence rather than an acute inflammatory response. This supports the hypothesis that persistent dermal immune activation may contribute to ongoing neuropathic pain. The observed effect was driven by a small subset of individuals and no significant differences were found between pain and no-pain subgroups. Nevertheless, macrophages can directly sensitise nociceptors via cytokines and growth factors, and their prolonged presence in cutaneous tissue has been associated with persistent pain states in both preclinical and clinical settings [48, 49].

Several limitations warrant consideration. The sample size, although adequate for an exploratory study, may have limited power to detect subtle group differences. Second, only basal conditions were assessed in vitro; fibroblasts may exhibit differential responses under inflammatory or metabolic stress, which were not modelled here. To minimise culture-induced senescence, we banked early-passage (passage 3 or 4) fibroblasts at ~80% confluence and used aliquots from this biobank under uniform conditions; nevertheless, we did not measure canonical senescence markers, which, given the links between fibroblast senescence, chronic low-grade inflammation and mitochondrial dysfunction [34, 35], remain a priority for future studies. Dermal macrophages were quantified solely by Iba1 staining, without additional markers to distinguish macrophage subsets or activation states, and lymphocyte populations were not assessed. While this approach provides a robust estimate of macrophage infiltration, it does not allow functional conclusions. Future studies will extend macrophage phenotyping beyond Iba1 using multiparameter immunostaining and transcriptomic approaches to define activation states, alongside lymphocyte profiling and spatial mapping of macrophage–nerve interactions; these analyses should clarify the dermal immune landscape in pDPN. Although our phenotyping included key fibroblast markers, future single-cell transcriptomics and spatial multiomics approaches may better resolve functional fibroblast states in situ.

In conclusion, our findings suggest that in the context of diabetic neuropathy, dermal fibroblasts do not show major alterations in subtype distribution, inflammatory output or mitochondrial function. However, fibroblast size correlated with neuropathy severity, indicating that subtle morphological changes may be relevant to disease progression. Nonetheless, the sample size was small and we cannot exclude that a larger sample could have detected differences. By contrast, dermal macrophage infiltration was elevated in pDPN, pointing to a persistent immune activation phenotype. These results refine current models of non-neuronal contributions to neuropathic pain and highlight the dermal immune microenvironment, rather than fibroblasts per se, as a promising therapeutic target in painful diabetic neuropathy. Nevertheless, the preserved mitochondrial phenotype in fibroblasts observed under basal conditions does not exclude the possibility that mitochondrial dysfunction may emerge under metabolic or inflammatory stress, or that other, non-tested phenotypes in fibroblasts could contribute to disease mechanisms. Future studies incorporating defined inflammatory or metabolic challenges will be important to determine whether stress-evoked fibroblast responses contribute to pDPN.

## Supplementary Information

Below is the link to the electronic supplementary material.

ESM Figures (PDF 1.51 MB)

## Data Availability

The datasets generated and/or analysed during the current study are available from the corresponding author on reasonable request.

## Abbreviations

CCCP: Carbonyl cyanide m-chlorophenyl hydrazone
CRP: C-reactive protein
CXCL8: C-X-C motif chemokine ligand 8
FAP: Fibroblast activation protein
FSP1: Fibroblast-specific protein 1
HSP60: Heat shock protein 60
Iba1: Ionised calcium-binding adaptor molecule 1
IENFD: Intraepidermal nerve fibre density
JC-1: 5,5′,6,6′-tetrachloro-1,1′,3,3′-tetraethylbenzimidazolylcarbocyanine iodide
MitoSOX: Mitochondrial superoxide indicator
MMP: Mitochondrial membrane potential
noDPN: Diabetes without diabetic polyneuropathy
npDPN: Pain-free diabetic polyneuropathy
NRS: Numeric Rating Scale
OCR: Oxygen consumption rate
pDPN: Painful diabetic polyneuropathy
PE: Phycoerythrin
PER: Proton efflux rate
ROS: Reactive oxygen species
TCNS: Toronto Clinical Neuropathy Score
TOMM20: Translocase of the outer mitochondrial membrane 20
