# Supplementary material for: Dermal fibroblast mitochondrial profiles in painful diabetic neuropathy
Source: Diabetologia. 2026 Jan 26;69(5):1354–69. doi: 10.1007/s00125-025-06660-8 (PMC13005869; doi:10.1007/s00125-025-06660-8)
Supplement: Supplementary file 1 — ESM Figures (PDF 1.51 MB) [file 125_2025_6660_MOESM1_ESM.pdf]

Electronic supplementary material

Dermal fibroblast mitochondrial profiles in painful diabetic neuropathy

Julie Mie Mølgaard Bentzen, Peter Kolind Brask-Thomsen, Maiken Krogsbæk, Xiaoli Hu, Jens Randel Nyengaard, Sandra Sif Gylfadottir, Pall Karlsson, Nanna Brix Finnerup, Rikke Katrine Jentoft Olsen, Zahra Nochi

Figures

ESM Fig. 1 Experimental workflow for fibroblast culture and skin immunohistochemistry.

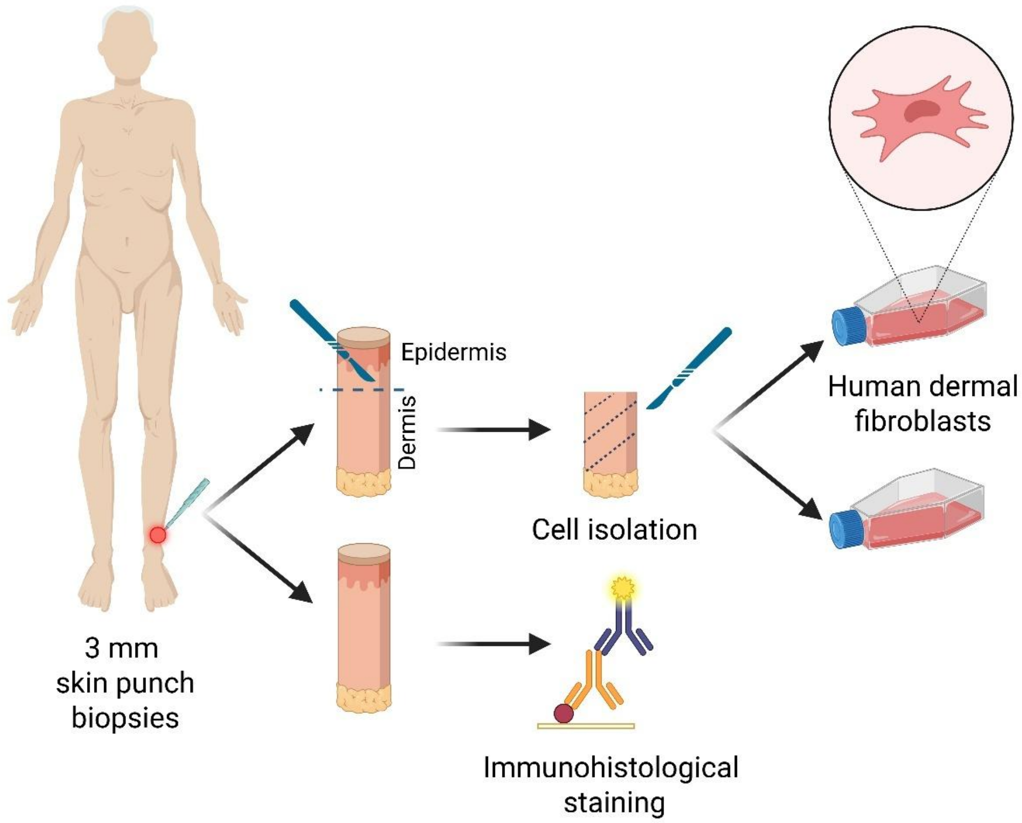

Two 3 mm punch biopsies were collected from the lower leg (10 cm above the lateral malleolus) of each participant. One biopsy was placed in supplemented RPMI medium and used to establish primary fibroblast cultures, which were expanded and cryopreserved for downstream analyses, including cell morphology, flow cytometry, inflammatory profiling, and mitochondrial assessments. The second biopsy was fixed in Zamboni's solution, cryoprotected in sucrose, and snap-frozen for subsequent immunohistochemistry (IHC), including quantification of intraepidermal nerve fibers, fibroblasts, mitochondria, Langerhans cells, and macrophages. Created with BioRender.com.

ESM Fig. 2 Flow cytometry gating strategy for fibroblast subpopulation analysis.

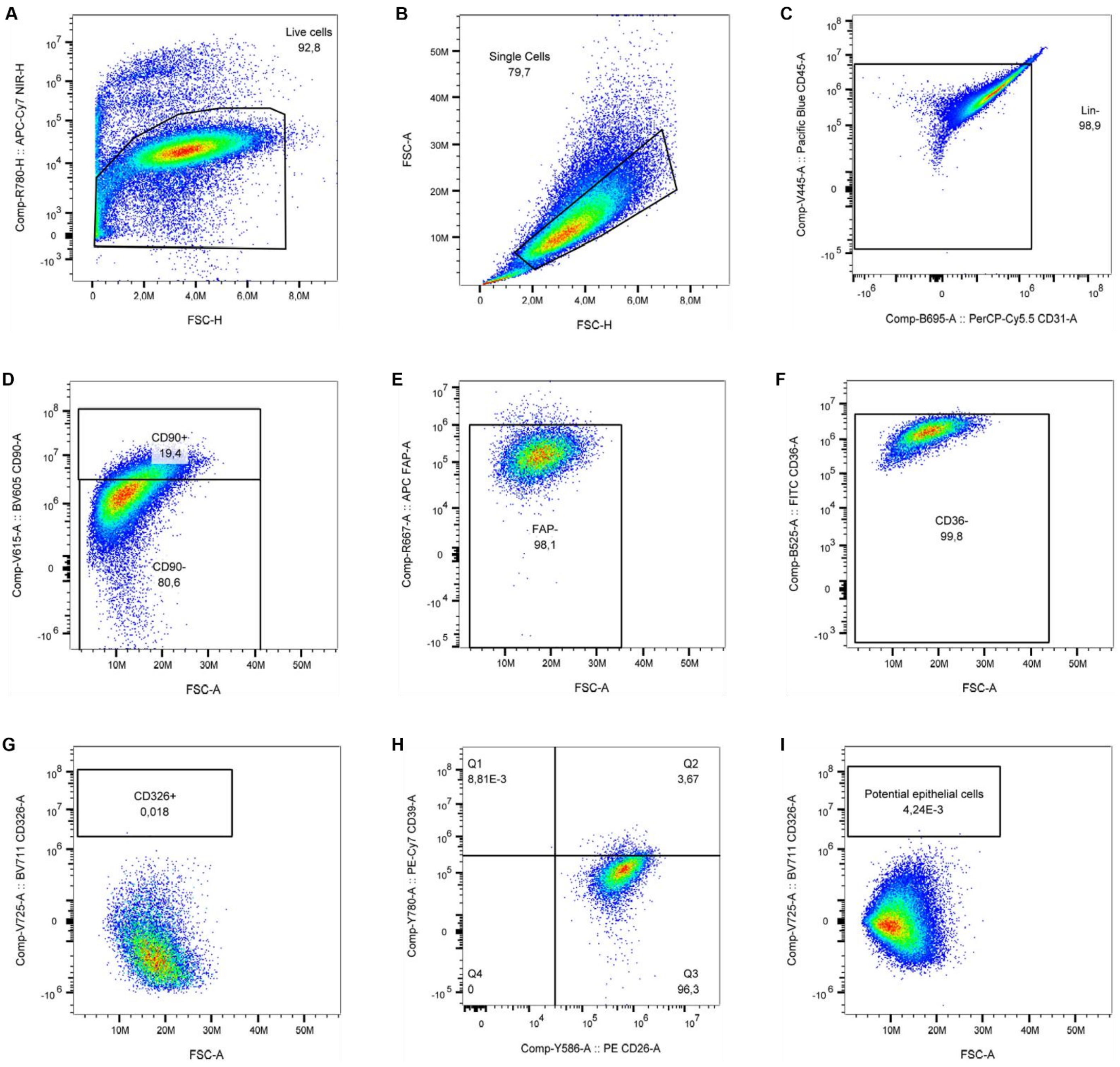

Single-cell suspensions from human dermal fibroblasts were sequentially gated to enrich for viable, non-hematopoietic, non-endothelial fibroblasts and assess their subtype composition. **(A)** Live cells (Zombie NIR-low), **(B)** Singlets, **(C)** lineage-negative ($CD45^{-}$ $CD31^{-}$) fibroblasts, **(D)** stratification into $CD90^{+}$ and $CD90^{-}$ fibroblast subsets. **(E)** FAP expression within $CD90^{+}$ fibroblasts, **(F)** CD36 expression within $CD90^{+}$ fibroblasts, **(G)** CD326 (EpCAM) expression within $CD90^{+}$ fibroblasts. **(H)** CD26 and CD39 co-expression within $CD90^{+}$ fibroblasts. **(I)** CD326 expression within $CD90^{-}$ fibroblasts to assess epithelial contamination. Marker analyses shown in panels (G–I) were performed for both $CD90^{+}$ and $CD90^{-}$ subsets; representative plots are shown for $CD90^{+}$ cells unless otherwise indicated.

ESM Fig. 3 Representative immunofluorescence staining of skin biopsy from a patient with pain-free DPN (npDPN).

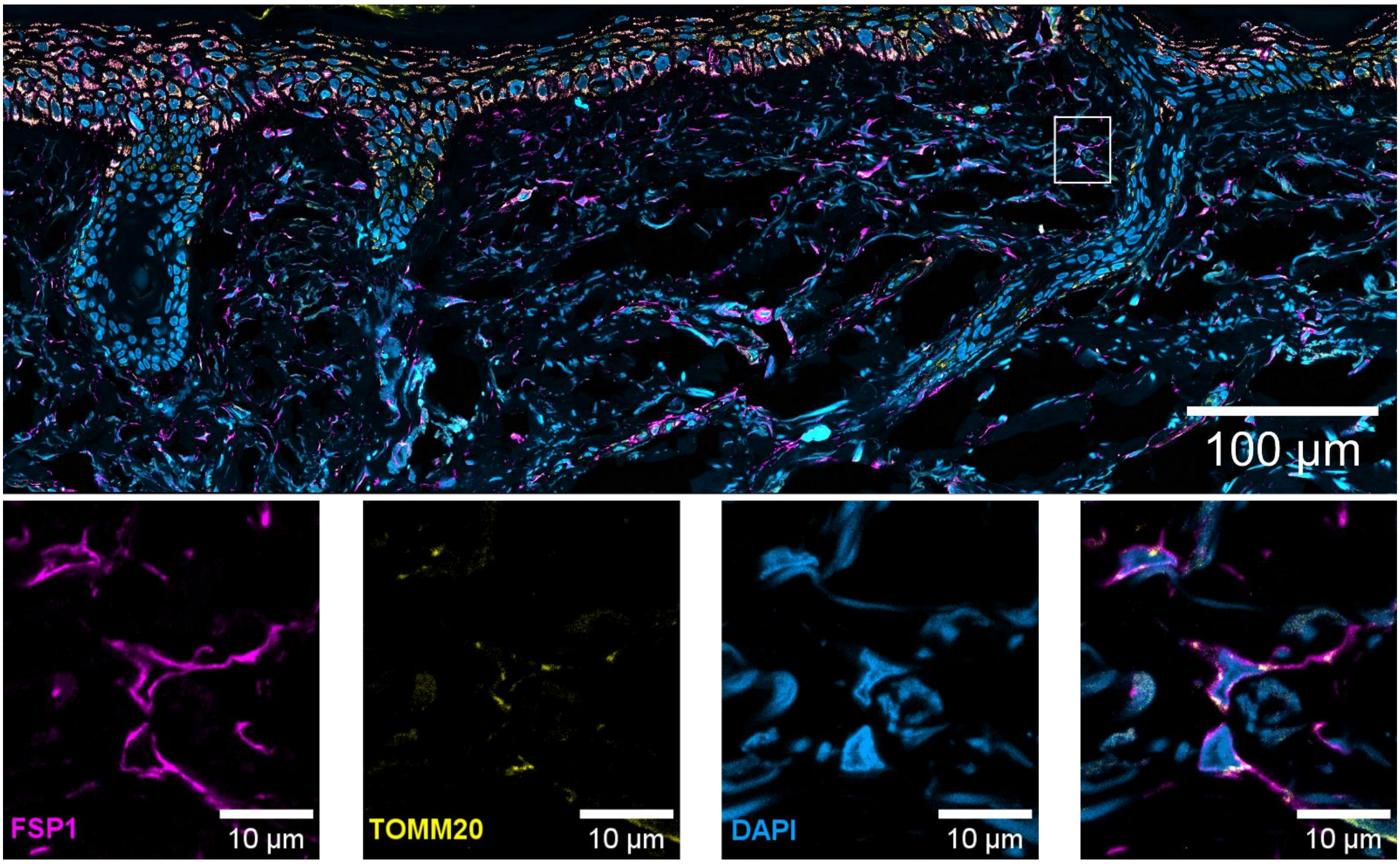

Whole-mount immunostaining shows cellular and subcellular structures in the epidermis and dermis. Nuclei are stained with DAPI (blue), fibroblasts are labeled using fibroblast-specific protein 1 (FSP1; magenta), and mitochondria are visualized with the outer mitochondrial membrane marker TOMM20 (yellow). The upper panel presents a low-magnification overview, illustrating the layered structure of the skin and the distribution of fibroblasts and mitochondria throughout the dermis. A white square indicates the area enlarged in the bottom panel. The bottom row shows high-magnification single-channel and merged images of the boxed region, highlighting fibroblast morphology and their associated mitochondrial network. Scale bars: 100 µm (overview) and 10 µm (high magnification).

ESM Fig. 4 Fibroblast cell size across individual participants.

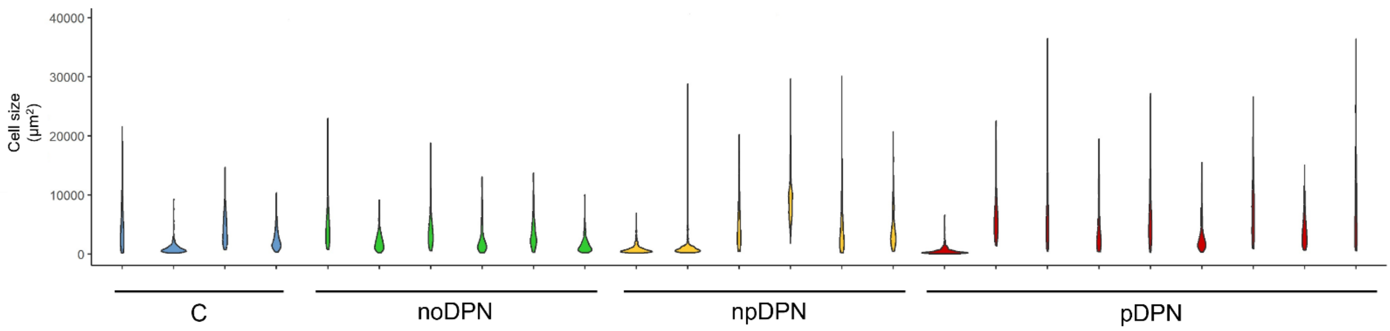

Violin plots showing the distribution of fibroblast cross-sectional area (μm²) across individual participants, grouped by clinical category: non-diabetic control participants (C), diabetes without polyneuropathy (noDPN), pain-free DPN (npDPN), and painful DPN (pDPN). Each violin represents one participant, and data reflect all fibroblasts quantified within that individual. Cell area was estimated using the unbiased 2D nucleator method from bright-field images acquired at ~80% confluence. One-way ANOVA demonstrated significant variability in cell size across participants (p<2.2e^{-16}).

ESM Fig. 5 Fibroblast subtype composition across clinical groups based on CD90, CD26, and CD39 expression.

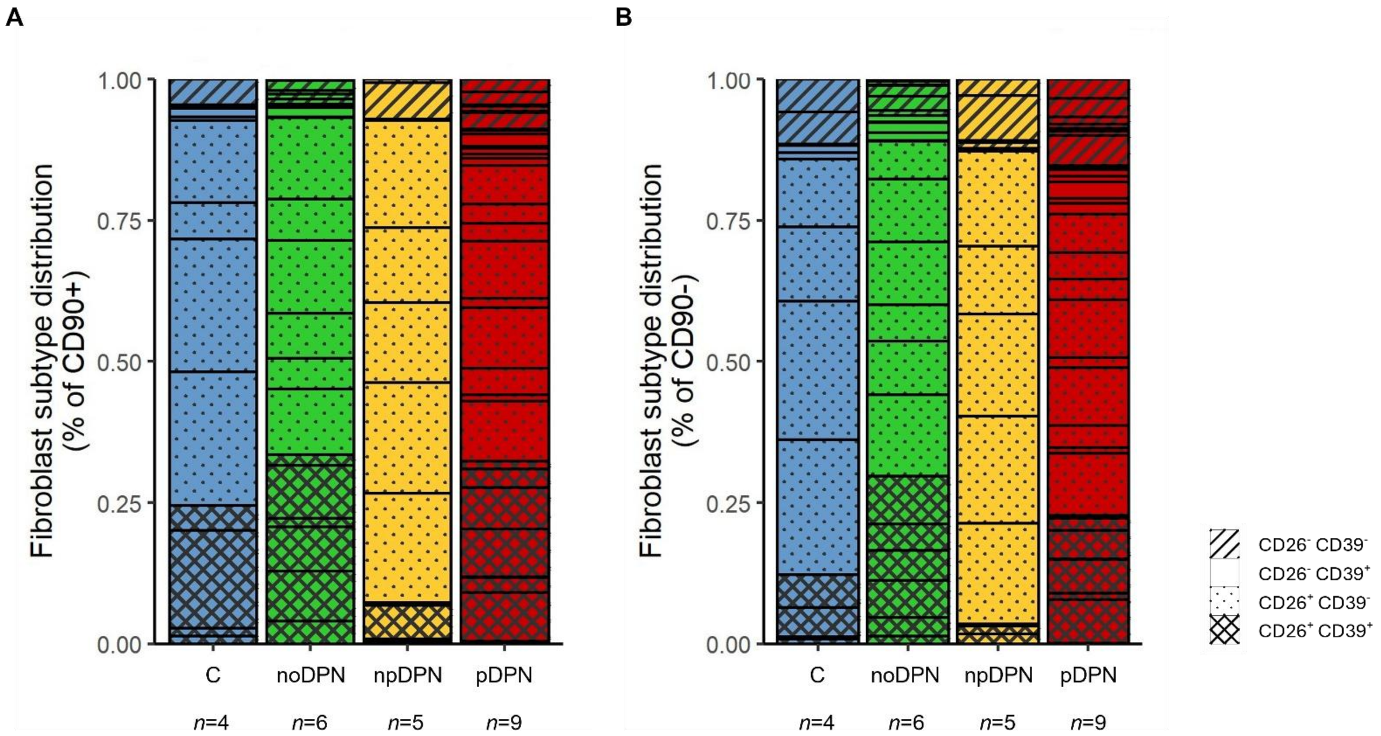

Distribution of fibroblast subtypes based on CD26 and CD39 co-expression within **(A)** $CD90^{+}$ and **(B)** $CD90^{-}$ fibroblast populations from control participants without diabetes (C), diabetes without diabetic polyneuropathy (noDPN), diabetes with pain-free DPN (npDPN), and diabetes with painful DPN (pDPN). Each stacked bar represents the proportional distribution (%) of fibroblast subtypes averaged across individuals within each clinical group. Statistical analyses revealed no significant differences among groups (Kruskal–Wallis test, all $p > 0.07$).

ESM Fig. 6 Proton efflux in dermal fibroblasts derived from non-diabetic controls and patients with diabetes with and without diabetic polyneuropathy, and pain.

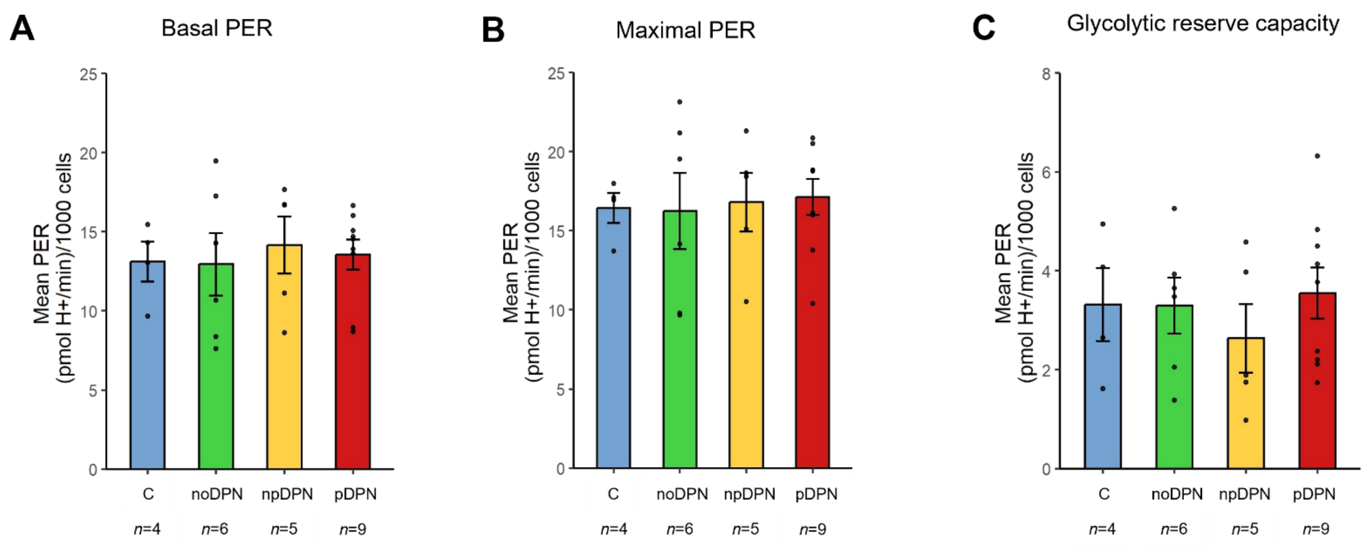

(A) Basal proton efflux rate (PER), **(B)** maximal PER, and **(C)** glycolytic reserve capacity were measured in fibroblasts derived from control participants without diabetes (C), diabetes without diabetic polyneuropathy (noDPN), diabetes with pain-free DPN (npDPN), and diabetes with painful DPN (pDPN). No statistically significant differences were observed across groups (ANOVA, all $p > 0.4$). Bars represent mean ± SD; each dot represents one individual.

ESM Fig. 7. Dermal fibroblast density, surface area, and mitochondrial characteristics in skin biopsies.

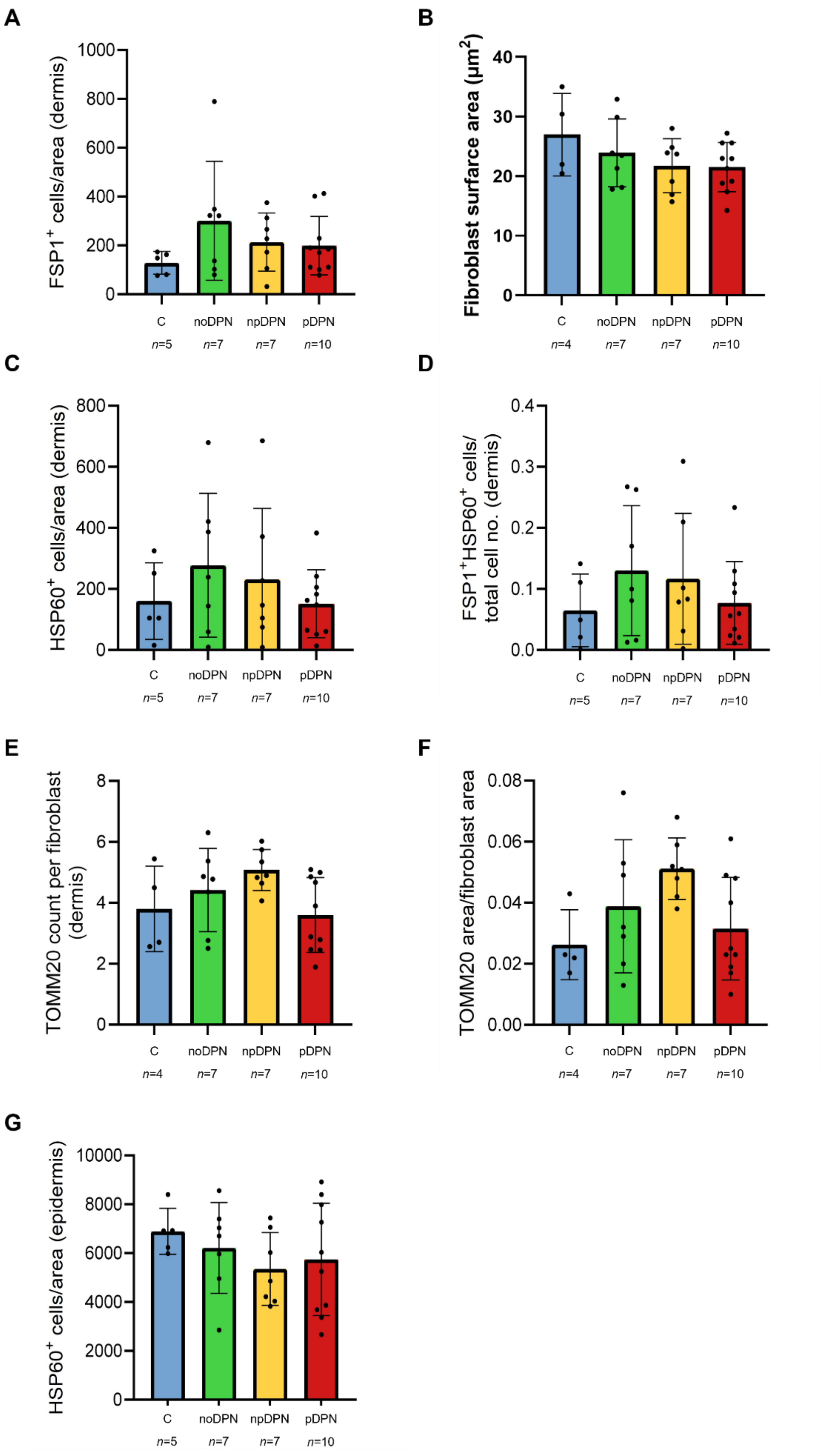

(A) Dermal fibroblast density assessed by FSP1⁺ cells per tissue area, **(B)** Mean fibroblast surface area measured per group, **(C)** Dermal HSP60⁺ cell density per tissue area, **(D)** Proportion of dermal cells co-expressing FSP1⁺ and HSP60⁺ relative to total cell count, **(E)** TOMM20 mitochondrial count per fibroblast, **(F)** TOMM20 mitochondrial area normalized to fibroblast area, **(G)** Epidermal mitochondrial density quantified as HSP60⁺ cells per epidermal area. Statistical comparisons performed by one-way ANOVA; no significant differences found ($p > 0.05$ for all comparisons). Bars represent mean ± SD; each dot represents one individual.
